# Diagnostic Accuracy and Predictive Factors of Sentinel Lymph Node Biopsy in Cutaneous Squamous Cell Carcinoma: A Systematic Review

**DOI:** 10.1177/22925503261491621

**Published:** 2026-09-30

**Authors:** Omar El Sewify, Marion Sylvain, Cory Tremblay, Helene Retrouvey, Ammara Ghumman

**Affiliations:** 1 Faculty of Medicine, 12369 Laval University, Quebec City, Canada; 2 Faculty of Medicine and Health Sciences, 12367 McGill University, Montreal, Canada; 3 Division of Plastic Surgery, 3710 McMaster University, Hamilton, Canada; 4 Division of Plastic, Reconstructive & Aesthetic Surgery, 7985 University of Toronto, Toronto, Canada

**Keywords:** Sentinel lymph node biopsy, SLNB, squamous cell carcinoma, SCC, skin cancer, staging, prognosis, diagnostic accuracy, biopsie du ganglion sentinelle, BGS, CE, carcinome épidermoïde, cancer de la peau, précision diagnostique, pronostic, stadification

## Abstract

**Introduction:**

Sentinel lymph node biopsy (SLNB) is increasingly used to stage cutaneous squamous cell carcinoma (cSCC), particularly in high-risk cases, though its diagnostic accuracy remains debated. This systematic review aims to evaluate SLNB detection rates, diagnostic accuracy, complications and predictors of positivity in patients with non-mucosal cSCC.

**Methods:**

Following PRISMA guidelines, Medline, Embase, Scopus and Cochrane Library were searched to January 2025. Studies reporting SLNB outcomes in histologically confirmed cSCC were included. Pediatric and mucosal/anogenital cases were excluded. Data on demographics, tumour characteristics, SLNB outcomes and complications were extracted. Comparisons between SLNB-positive, negative and false-negative groups were performed (*p* < 0.05).

**Results:**

Forty-four studies (3043 patients, 3050 lesions) were included. Among 2956 patients undergoing SLNB, nodes were identified in 96.9%, with positivity in 10.9%. False negatives occurred in 1.7% of the cases (rate 13.2%). SLNB-positive patients had larger tumours (4.24 vs 2.88 cm, *p* < 0.0001) and deeper invasion (9.81 vs 4.75 mm, *p* = 0.0007), higher rates of perineural invasion (*p* = 0.0436), poor differentiation (*p* = 0.009) and more T2 disease (*p* = 0.04). No significant differences were found for sex (*p* = 0.5), immunosuppression (*p* = 0.068) or tumour location (*p* = 0.1), though immunosuppression was more prevalent in SLNB-positive cases. Recurrence (24.3% vs 4.2%) and disease-specific mortality (18.2% vs 3.7%) were higher in SLNB-positive patients (*p* < 0.0002). Complication rates were low (3.2%).

**Conclusion:**

SLNB demonstrates high detection but notable false negative rates in cSCC. Positive SLNB correlates with aggressive tumour features and worse outcomes. SLNB may assist staging in high-risk patients but should be used selectively, with close surveillance in SLNB-negative cases.

## Résumé

**Introduction:**

La biopsie du ganglion sentinelle (BGS) sert de plus à stadifier le carcinome épidermoïde cutané (CEC), notamment dans les cas à haut risque, mais sa précision diagnostique demeure contestée. La présente analyse systématique vise à évaluer les taux de détection favorisés par la BGS, sa précision diagnostique, ses complications et les facteurs prédictifs de résultats positifs chez les patients ayant un CEC non mucineux.

**Méthodologie:**

Conformément aux lignes directrices PRISMA, les chercheurs ont fouillé les bases de données Medline, Embase, Scopus et de la Bibliothèque Cochrane jusqu’en janvier 2025. Ils ont conservé les études qui rendaient compte des résultats de la BGS chez des patients ayant un CEC confirmé sur le plan histologique et exclu les cas pédiatriques et les atteintes muqueuses ou anogénitales. Ils ont extrait les données démographiques, les caractéristiques des tumeurs, les résultats de la BGS et les complications. Ils ont également comparé les groupes ayant obtenu des résultats de la BGS positifs, négatifs et faussement négatifs (*p* < 0.05).

**Résultats:**

Au total, 44 études (3043 patients, 3050 lésions) ont été retenues. Chez les 2956 patients qui avaient subi une BGS, 96,9% avaient des ganglions, et 10,9% ont obtenu des résultats positifs. Les résultats étaient faussement négatifs dans 1,7% des cas (taux de 13,2%). Les patients dont les résultats de la BGS étaient positifs présentaient des tumeurs plus volumineuses (4.24 cm par rapport à 2,88 cm, *p* < 0.0001) et une invasion plus profonde (9.81 mm par rapport à 4,75 mm, *p* = 0.000 7), des taux plus élevés d’invasion périneurale (*p* = 0.043 6), une mauvaise différenciation (*p* = 0.009) et une plus grande prévalence de carcinome de stade T2 (*p* = 0.04). Les chercheurs n’ont observé aucune différence en fonction du sexe (*p* = 0.5), de l’immunosuppression (*p* = 0.068) ou de l’emplacement de la tumeur (*p* = 0.1), même si l’immunosuppression était plus fréquente lorsque les résultats de la BGS étaient positifs. Les taux de récidive (24,3% par rapport à 4,2%) et de létalité (18,2% par rapport à 3,7%) étaient plus élevés chez les patients dont les résultats de la BGS étaient positifs (*p* < 0.0002). Les taux de complications étaient faibles (3,2%).

**Conclusion:**

La BGS favorise des taux de détection élevés, mais les résultats faussement négatifs de CEC sont appréciables. Des résultats positifs à la BGS sont corrélés à des caractéristiques tumorales plus agressives et à de moins bons pronostics. La BGS peut contribuer à la stadification chez les patients à haut risque, mais devrait être utilisée de façon sélective et donner lieu à une surveillance étroite lorsque les résultats de la BGS sont négatifs.

## Introduction

Cutaneous squamous cell carcinoma (cSCC) is the second most common type of skin cancer globally, and its incidence continues to increase.^1‐3^ It represents a growing public health concern, particularly among aging populations and immunosuppressed individuals.^1‐3^ Most cSCCs develop on areas often exposed to the sun, such as the head, neck and limbs.^4^ While the majority of cases are curable with local excision and have excellent prognosis, a subset of tumours presents with high-risk features that increase the chances of lymph node involvement, recurrence and disease-specific mortality.^1,5^ These high-risk characteristics include large tumour size, deeper invasion, perineural or lymphovascular involvement and poor histologic differentiation.^6,7^

Sentinel lymph node biopsy (SLNB) is increasingly being used to help stage and guide treatment decisions in patients with high-risk cSCC.^8,9^ In melanoma, SLNB is a well-established standard of care as it plays a key role in identifying nodal involvement and guiding adjuvant treatment decisions.^10^ However, the role of SLNB in cSCC remains less clearly defined.^5^ The diagnostic accuracy, false negative rates and overall clinical utility of SLNB in this setting continue to be debated.^11^ As a result, practice varies widely across institutions.

Existing reviews on SLNB in cSCC are scarce, and those available are dated and methodologically limited. These include small sample sizes, inconsistent inclusion criteria and wide variability in how outcomes are reported.^12^ A particular issue with many earlier studies is the inclusion of tumours from mucosal sites, such as the lips and anogenital region, which behave differently from purely cutaneous lesions.^13^ These anatomical subsites are now recognized to have distinct biological behaviours, different lymphatic drainage patterns and often require separate staging systems and treatment approaches.^14^ Mucosal SCCs tend to be more aggressive and are associated with higher rates of metastasis compared to their purely cutaneous counterparts.^13^ Their inclusion in past analyses likely contributed to variability in outcomes and limited the generalizability of findings to patients with cutaneous SCC.

Given the increasing use of SLNB in clinical practice and the evolution of evidence over the past decade, there is a pressing need for a contemporary systematic review focusing exclusively on cutaneous, non-mucosal SCC. By excluding the majority of mucosal sites, we aim to reduce clinical heterogeneity and better characterize the role of SLNB within a more uniform population. This review synthesizes current evidence on SLNB performance in non-mucosal cSCC, including detection rates, diagnostic accuracy (with attention to false negative cases), complication rates and histopathologic features associated with SLNB positivity. Through this focused lens, we aim to clarify the potential benefits and limitations of SLNB and guide its use in high-risk cSCC management.

## Materials and Methods

This systematic review was conducted in accordance with the 2020 Preferred Reporting Items for Systematic Reviews and Meta-Analyses (PRISMA) guidelines.^15^ The population consisted of patients diagnosed with cSCC. There were no limitations with respect to country of publication. The protocol for this systematic review was registered on Open Science Framework (osf.io/5jvds).

### Search Strategy

A comprehensive search of the Medline (via Ovid), Embase (via Ovid), Scopus and Cochrane Library databases was performed from inception up until January 2025 to identify studies reporting outcomes of SLNB in patients with histologically confirmed cSCC. The search strategy was designed and applied to the different databases. Citations were uploaded into EndNoteTM X9 (Clarivate Analytics, Philadelphia, PA, USA). Following deduplication, references were uploaded into Covidence systematic review software (Veritas Health Innovation, Melbourne, VIC, Australia), an online systematic review software. Both title and abstract screening, as well as full-text review were performed by two independent reviewers (OE, MS). Any disagreements between reviewers were resolved through consensus or discussion with a third reviewer (AG).

### Study Eligibility

Strict inclusion and exclusion criteria were established. Included study types encompassed randomized controlled trials, prospective and retrospective studies, case-control studies and case series. Systematic reviews, meta-analyses, letters to the editor, viewpoints, commentaries, case reports, cadaveric studies, incomplete abstracts and non-English studies were excluded. Studies with and without a comparator group were eligible for inclusion. Pediatric (<18 years old) patients were excluded. Adult patients (≥18 years old) who underwent SLNB were eligible for inclusion. Finally, studies incorporating terms interchangeable with ‘cSCC’ and ‘SLNB’ for non-mucosal cutaneous SCC were included.

### Data Extraction and Synthesis

Data extraction was performed by two reviewers using Microsoft Excel for Mac, Version 16.92 (Microsoft Corporation, Redmond, WA, USA). Any disagreements were resolved through consensus with the principal investigator. Data points extracted included study characteristics (title, authors, year, study design, funding and country of publication), patient demographics (number of patients, sex, age, immunosuppression status), tumour characteristics (size, depth, location, differentiation, perineural invasion (PNI), lymphovascular invasion (LVI)) and outcomes (SLNB results, recurrence mortality and procedure-related complications, follow-up time). Extracted data were provided in summary tables (Supplemental Tables S1–S3, Tables 1‐4).

**Table 1.** Summary of Patient Characteristics.

General Characteristics	Values	Patients (*n*)	Tumours (*n*)
Total number of patients	3043	—	—
Total number of cSCCs	3050	—	—
Mean age (range) in years	70.72 (15–102)	1657	NA
Sex (%)	M: 2067 (71.8)	2878	NA
	F: 811 (28.2)		
Immunodeficiency (%)	285 (26.2)	1086	NA
SLNB-related complications (%)	16 (3.2)	506	NA
Dehiscence	2 (9.1)	22	
Surgical site infection	4 (2.3)	173	
Hematoma	2 (1.2)	173	
Transient face palsy	1 (1.4)	74	
Lymphedema	0 (0.0)	5	
Allergic reaction	0 (0.0)	8	
Bacterial lymphangitis	1 (2.0)	49	
Lymphorrhea	1 (2.0)	49	
Hemorrhage	1 (2.0)	49	
Seroma	1 (2.7)	37	
Others	3 (4.7)	64	
Deaths (%)	156 (15.9)	979	NA
SCC-related	54 (5.5)	979	
SLNB-positive	6 (18.2)	33	
SLNB-negative	16 (3.7)	430	
False negative	5 (45.5)	11	
Recurrence (%)	163 (13.1)	NA	1244
Local	40 (6.1)		655
Regional	13 (2.0)		655
SLNB-positive	9 (24.3)		37
SLNB-negative	19 (4.2)		457
False negative	0 (0.0)		2
Mean follow-up (range) in months	35.3 (0.5–180)	1168	NA

NA: not applicable, cSCC: cutaneous squamous cell carcinoma, SLNB: sentinel lymph node biopsy.

**Table 2.** Summary of Tumour Characteristics.

General Characteristics	Values	Tumours (*n*)
Location (%)		2684
Head and neck	2274 (84.7)	
Non-head and neck	410 (15.3)	
Mean diameter (range) (cm)	2.78 (0.29–30)	725
Mean depth (range) (mm)	6.01 (0.5–35)	856
Recurrent tumour (%)	131 (19.0)	689
High-risk features (%)		2688
High-risk	2541 (94.5)	
Low-risk	147 (5.5)	
Histological differentiation (%)		2179
In-situ	4 (0.2)	
Well differentiated	467 (21.4)	
Moderately differentiated	556 (25.5)	
Poorly differentiated	1058 (48.6)	
Undifferentiated	90 (4.1)	
Sarcomatoid	4 (0.2)	
Invasion (%)		
PNI	774 (37.7)	2054
LVI	28 (6.7)	421
Area of chronic inflammation* (%)	130 (28.6)	455

Cm: centimeter, LVI: lymphovascular invasion, mm: millimeter, PNI: perineural invasion.

*Including scars, radiation dermatitis, osteomyelitis, ulceration, and other chronic inflammatory processes.

**Table 3.** SLNB Results.

Measure	Tumours (*n*/*N*)	Percentage (%)
Total SLNB performed (%)	2956/3050	96.9
Successful SLN identification (%)	2864/2956	96.9
SLNB-positive cases (%)	318/2909	10.9
SLNB-negative cases (%)	2591/2909	89.1
False negative SLNBs (%)	48/2909	1.7
False negative rate (FN/[TP + FN])	48/(318 + 48)	13.2

FN: false negative, SLN: sentinel lymph node, SLNB: sentinel lymph node biopsy, TP: true positive.

**Table 4.** Comparison of Clinical and Pathological Features between SLNB-Positive, SLNB-Negative, and False-Negative cSCC Cases.

Measure	SLNB-Positive (*n*)	SLNB-Negative (*n*)	FN (*n*)	− Versus –	− Versus FN
				*p*-value	*p*-value
Sex (%)					
Male	23 (60.5)	248 (66.0)	10 (71.4)	0.5022	0.6710
Female	15 (39.5)	128 (34.0)	4 (28.6)		
Mean age (years)	67.7 (*n* = 38)	69.9 (*n* = 335)	65.6 (*n* = 11)	—	—
Mean tumour diameter (cm)	4.24 (*n* = 18) (SD 2.42)	2.88 (*n* = 214) (SD 0.60)	—	**<0**.**0001**	—
Mean tumour thickness (mm)	9.81 (*n* = 14) (SD 4.05)	4.75 (*n* = 149) (SD 5.32)	—	**0**.**0007**	—
PNI (%)					
Present	11 (44.0)	67 (25.3)	4 (36.4)	**0**.**0436**	0.4100
Absent	14 (56.0)	198 (74.7)	7 (63.6)		
Immunosuppressed (%)					
Yes	16 (41.0)	69 (26.8)	3 (50.0)	0.0682	0.2087
No	23 (59.0)	188 (73.2)	3 (50.0)		
Tumour location (%)					
HN	27 (61.4)	381 (72.8)	12 (85.7)	0.1033	0.2836
Non-HN	17 (38.6)	142 (27.2)	2 (14.3)		
Differentiation (%)					
Well differentiated	5 (25.0)	128 (48.5)	0 (0.0)	WM versus PU	0.07
Moderately differentiated	9 (45.0)	97 (36.7)	5 (83.3)	W versus P	**0.009**
Poorly differentiated	6 (30.0)	33 (12.5)	1 (16.7)	W versus MPU	**0.04**
Undifferentiated	0 (0.0)	6 (2.3)	0 (0.0)		
Stage*					
T1 (%)	2 (6.4)	35 (18.1)	—	T1 versus T2–4	0.1
T2 (%)	14 (45.2)	54 (28.0)	—	T1-2versus T3-4	0.6
T3 (%)	15 (48.4)	97 (50.3)	—	T1 versus T3	0.2
T4 (%)	0 (0.0)	7 (3.6)	—	T1 versus T2	**0.04**
High-risk tumours					
Yes (%)	75 (100.0) (SD 0)	697 (91.6) (SD 2.8)	16 (100.0)	**0**.**0001**	**0.0001**
No (%)	0 (0.0)	64 (8.4)	0 (0.0)		
Deaths (%)					
SCC-related death	6 (18.2)	16 (3.7)	5 (45.5)	**0**.**0002**	**<0.0001**
Alive or non-SCC death	27 (81.8)	414 (96.3)	11 (54.5)		
Recurrence (%)					
Yes	9 (24.3)	19 (4.2)	0 (0.0)	**<0.00001**	—
No	28 (75.7)	438 (95.8)	2 (100.0)		

cm: centimeter, cSCC: cutaneous squamous cell carcinoma, FN: false negative, HN: head and neck, mm: millimeter, MPU: moderately, poorly or undifferentiated, P: poorly differentiated, PU: poorly or undifferentiated, SCC: squamous cell carcinoma, SLNB: sentinel lymph node biopsy, W: well differentiated, WM: well or moderately differentiated, +: SLNB-positive group, −: SLNB-negative group.

*AJCC Version 9 Cancer Staging System.

### Statistical Analysis

Data was pooled using means and weighted averages where applicable. Outcomes of interest included SLNB detection rate, false negative rate and the relationship between SLNB status and pathological features. We compared SLNB-positive, SLNB-negative and false negative groups using chi-square tests for categorical data and unpaired t-tests for continuous data. A *p*-value of <0.05 was considered statistically significant.

## Results

### Study Characteristics

A total of 44 studies published between 2003 and 2024 were included in this systematic review, representing data from 15 countries, with the United States (*n* = 13) and Japan (*n* = 10) contributing to the majority of studies (Supplemental Table S1).

### Risk of Bias Assessment

Risk of bias was assessed using two different tools, selected according to study design, as outlined in Supplemental Table S4. For the single randomized controlled trial, Spyropoulou et al,^12^ the RoB2 assessment indicated an overall high risk of bias. The high risk was primarily driven by ‘high risk’ concerns related to deviations from intended interventions, alongside with ‘some concerns’ regarding randomization and the measurement of outcomes. For non-randomized studies, the MINORs (Methodological Index for Non-Randomized Studies) evaluation was applied. Among the 13 comparative studies, MINORs scores ranged from 16 to 21 out of a maximum of 24, with most studies lacking a prospective sample-size calculation and blinded endpoint assessment. Common areas contributing to bias in these studies included a lack of prospective calculation of study size, and varied performance on baseline equivalence of groups. In the 25 non-comparative studies, MINORs scores varied more widely (6–15 out of 16), reflecting inconsistent prospective data collection, variable follow-up completeness and incomplete reporting of losses to follow-up. Taken together, these assessments highlight moderate methodological quality overall and show the need to interpret pooled outcomes with caution.

### Patient Demographics and Tumour Characteristics

Across all included studies, 3043 patients with 3050 cSCCs were reported. The mean follow-up duration was 35.3 months (range: 0.5–180 months; *n* = 1168 patients, 25 studies). Among 2878 patients for whom patient sex or gender was reported, 71.8% were male and 28.2% were female. The mean age was 70.7 years (range: 15–102; *n* = 1657 patients, 24 studies) (Supplemental Table S2, Table 1).

Immunosuppression was present in 26.2% of patients (*n* = 285/1086), with 14% having undergone solid organ transplantation (*n* = 82/587). Tumour location was primarily in the head and neck region (84.7%, *n* = 2274/2684 cSCCs), with 15.3% involving non-head and neck sites. The mean tumour size was 2.78 cm (range: 0.29–30 cm; *n* = 725 cSCCs), and 94.5% (*n* = 2541/2688 cSCCs) were classified as high-risk based on study-defined criteria. Mean tumour depth was 6.01 mm (range: 0.5–35, *n* = 856 cSCCs). Among 2179 tumours, 0.2% were in-situ, 21.4% were well differentiated, 25.5% were moderately differentiated, 48.6% were poorly differentiated, 4.1% were undifferentiated and 0.2% were sarcomatous. PNI was reported in 37.7% of cases (*n* = 774/2054 cSCCs) and LVI was found in 6.7% (*n* = 28/421 cSCCs). 130 of 455 tumours were arising from areas of chronic inflammation (Supplemental Tables S2 and S3, Tables 1 and 2).

A total of 156 deaths were reported (15.9%, *n* = 156/979 patients), where 54 were SCC-related (5.5%, *n* = 54/979 patients). About 163 tumours recurred (13.1%, *n* = 163/1244 cSCCs), with 40 being local (6.1%, *n* = 40/655 cSCCs) and 13 being regional (2.0%, *n* = 13/655 cSCCs) (Table 1).

### Sentinel Lymph Node Biopsy (SLNB) Outcomes

A total of 2956 patients underwent SLNB (96.6%). Sentinel nodes were successfully identified in 2864 patients, yielding a detection rate of 96.9% and a failed identification rate of 3.1%. Among 2909 evaluable cases, SLNB was positive in 318 (10.9%) and negative in 2591 (89.1%). A total of 48 false negatives were reported (1.7% of SLNBs), corresponding to a false negative rate of 13.2%, calculated as false negatives ÷ (true positives + false negatives) (Table 3).

SLNB-related complications were rare, with a reported complication rate of 3.2% (*n* = 16/506; SD = 0.0077), with the most common complications being surgical site infection (*n* = 4/16), dehiscence (*n* = 2/16) and hematoma (*n* = 2/16) (Table 1). Clinically important long-term complications were infrequently reported. Among two studies (34 patients) that specifically documented facial nerve outcomes, one transient facial nerve palsy occurred (2.9%), with no permanent deficits. Lymphedema was explicitly assessed in only one study (five patients), with no reported cases. Specific injuries to the marginal mandibular, greater auricular or intercostobrachial nerves were not separately described.

### Comparative Analysis of SLNB-Positive and SLNB-Negative Populations

Patients with positive SLNB results had more aggressive tumour features than those with negative results. The average tumour diameter in the SLNB-positive group was larger, measuring 4.24 cm (SD 2.42) versus 2.88 cm (SD 0.60) in the negative group. This difference was statistically significant at *p* < 0.0001. Tumour thickness was significantly greater in the SLNB-positive group, with a mean of 9.81 mm (SD 4.05) compared to 4.75 mm (SD 5.32) in the SLNB-negative group (*p* = 0.0007), indicating deeper local invasion in patients with nodal metastasis (Table 4).

Histologically, poorly differentiated tumours were significantly more common among SLNB-positive patients than well-differentiated tumours (*p* = 0.009). Furthermore, tumours with lower overall degrees of differentiation were significantly more frequent in the SLNB-positive group than in the SLNB-negative group (*p* = 0.04). Furthermore, PNI was observed more often among SLNB-positive tumours (44.0% vs 25.3%, *p* = 0.0436), suggesting an association between neural invasion and nodal spread. The SLNB-positive group had significantly more stage T2 than T1 tumours when compared to the SLNB-negative group (*p* = 0.04) (Table 4).

Tumours with high-risk features were more commonly seen in SLNB-positive cases with 100% (SD 0%) rate compared to 91.6% (SD 2.8%) in SLNB-negative cases (*p* = 0.0001). The SLNB-positive group had significantly higher mortality rates, at 18.2% (*n* = 6/33) versus 3.7% (*n* = 16/430) (*p* = 0.0002), and recurrence, at 24.3% (*n* = 9/37) versus 4.2% (*n* = 19/457) (*p* < 0.00001) (Table 4).

No statistically significant differences were found in patient sex (*p* = 0.5), immunosuppression status (*p* = 0.68) or tumour location (*p* = 0.1). Notably, immunosuppression was more prevalent among SLNB-positive patients (41.0%, *n* = 16/39) compared to the SLNB-negative group (26.8%, *n* = 69/257) (Table 4).

### False Negative Cases

Among patients with false negative SLNBs, 71.4% were male (*n* = 10/14). The mean age was 65.6 years (*n* = 11). PNI was reported in 36.4% (*n* = 4/11), and 50% (*n* = 3/6) were immunosuppressed. Both rates are higher than the SLNB-negative group, but not statistically significant (*p* = 0.4, *p* = 0.2). All tumours were high risk (*n* = 16/16), and 85.7% (*n* = 12/14) were located in the head and neck region. When comparing the high-risk rate to the SLNB-negative group, the difference was statistically significant (*p* = 0.0001), with false negative cases presenting more high-risk features. None of the false negative cases were associated with well-differentiated tumours; most were moderately (*n* = 5) or poorly (*n* = 1) differentiated. Compared to SLNB-negative cases, false negative cases had a significantly higher SCC-related mortality at 45.5% (*n* = 5/16) compared to 3.7% (*n* = 16/430) (*p* < 0.0001) (Table 4).

## Discussion

This systematic review provides an updated and focused synthesis of SLNB outcomes in patients with cSCC. Our findings demonstrate that SLNB is both feasible and safe, with a high detection rate (96.9%) and low complication rate (3.2%). However, a false negative rate of 13.2% reminds us that a negative SLNB does not guarantee the absence of occult metastasis.

Notably, SLNB positivity was significantly associated with larger tumour size, greater depth of invasion, poor differentiation, and PNI. In fact, our pooled data resonate with prior case series portraying SLNB positivity in roughly 10% to 15% of high-risk cSCCs, with the odds rising when depth exceeds 6 mm or when histology is poorly differentiated.^16,17^ Furthermore, SLNB-positive patients experienced markedly higher recurrence (24.3%) and disease-specific mortality rates (18.2%), compared to their SLNB-negative counterparts (4.2% recurrence and 3.7% mortality). These findings reinforce SLNB's potential role as a prognostic tool in select high-risk cases, while also highlighting the need for cautious interpretation of negative results.

Our findings are consistent with, but expand upon, past evidence in the field. In a systematic review published in 2006, Ross et al found a SLNB-positive rate of 24% in anogenital and 21% in nonanogenital patients.^18^ Tejera-Vaquerizo et al specifically looked at positive rates in different stages. Based on the AJCC-8 criteria, patients with T2 staging had a SLNB-positive rate of 8.3%, compared to 25% for stage T3.^19^ While earlier studies, such as Renzi et al,^20^ attempted to evaluate SLNB in cSCC, many of their studies were published over a decade ago, included mucosal lesions, and were limited by small sample sizes and heterogeneous methodologies. In a study looking at SCC of the lip and oral cavity, Matos et al showed a rate of occult metastasis of 23.1%, which is much higher than our SLNB-positive rate of 10.9%.^21^ In contrast, our review excluded mucosal and anogenital SCCs to reduce clinical heterogeneity, reflecting the current consensus that these subsites possess distinct biological behaviours, lymphatic drainage patterns and staging paradigms. This distinction is clinically meaningful with inclusion of aggressive mucosal tumours in earlier reviews likely inflating SLNB positivity and recurrence rates, making it difficult to assess its true utility in purely cutaneous disease.^18,20,21^

Importantly, this review affirms that SLNB positivity is tightly linked to biologically aggressive tumour characteristics. The statistically significant associations observed with larger tumour diameter, increased thickness, poor differentiation and PNI support the selective use of SLNB in high-risk patients. These pathological features are already embedded in NCCN and AJCC high-risk stratification guidelines and may serve as key determinants for SLNB eligibility.^22^

Although institutional practice remains heterogeneous, the studies included in this review illustrate how SLNB selection varies across centers. Some institutions limited SLNB to head and neck cSCC, whereas others focused on high-thickness tumours, institution-defined high-risk lesions or specific anatomical subsites such as the upper extremity or periocular region. This variability reflects the absence of standardized eligibility criteria despite broad agreement that SLNB should be reserved for patients with clinically node-negative disease and multiple high-risk features. Consequently, patient selection is often individualized and guided by multidisciplinary assessment rather than a universally accepted protocol.

Current guidelines similarly reflect the uncertainty surrounding SNLB in cSCC. Alberta Health Services does not recommend SLNB routinely for high-risk cSCC, although consideration may be given to selected clinically and radiologically node-negative patients with multiple high-risk features. NCCN similarly supports discussion and consideration of SLNB in selected patients with very-high-risk cSCC and a clinically normal nodal basin, while the Academy of Dermatology concludes that the value of SLNB in cSCC remains uncertain.^22,23^ The findings of the present review are consistent with this cautious approach: although SLNB demonstrated a high detection rate and low morbidity, the 13.2% false negative rate, heterogeneity of the available evidence and absence of evidence demonstrating improved survival preclude recommending routine SLNB based on the current literature.

Conversely, we found no statistically significant association between SLNB positivity and patient sex, immunosuppression or tumour location. However, the prevalence of immunosuppression was higher among SLNB-positive patients (41.0% vs 26.8%), which may have failed to reach significance due to small sample size or inconsistent reporting. This reflects findings from transplant recipients, in which UV-driven tumuors exhibit more aggressive behaviours due to their state of chronic immunosuppression.^17,24^ This supports the potential value of closer nodal surveillance in the immunosuppressed population, particularly solid organ transplant recipients.^17,24^

Assessing the accuracy of SLNB is particularly important because a false-negative result may compromise the recognition and treatment of regional metastatic disease. Several factors may contribute to false negative SLNBs in cSCC. Most tumours in the present review arose in the head and neck, were complex, variable and occasionally bilateral lymphatic drainage can complicate the identification of the true sentinel node. Prior excision, scar formation, tumour-related lymphatic obstruction, failure or limited migration of radiotracer, and variation in tracer selection, injection technique, preoperative lymphoscintigraphy and intraoperative node identification may further impair mapping accuracy. False negatives may also arise from incomplete pathological assessment, particularly when serial sectioning, immunohistochemistry or other ultrastaging techniques are not routinely used. Although these mechanisms may plausibly contribute to the observed 13.2% false negative rate, the available aggregate data did not permit identification of the relative contribution of the tumour location, mapping failure or pathological technique.^18,25^

The clinical consequences of the false negative SLNBs may be substantial. In this review, patients subsequently classified as false negative had significantly greater cSCC-related mortality than those who remained SLNB negative; however, the included studies did not consistently report timing, extent, resectability or morbidity of subsequent regional recurrences. It was therefore not possible to determine whether these patients developed unresectable nodal disease, required more extensive lymphadenectomy or radiation, or experienced treatment-related morbidity that might have been avoided through earlier nodal detection. These reporting limitations are particularly relevant as adjuvant systemic therapy evolves. The phase III C-POST trial demonstrated improved disease-free survival with adjuvant cemiplimab in patients with high-risk cSCC following surgery and radiation, and cemiplimab is now approved in the United States for this indication.^26^ However, this does not establish that patients with a positive SLNB alone would necessarily meet criteria for adjuvant treatment, because eligibility depends on the overall pathological and treatment context. Nevertheless, a false negative SLNB could theoretically delay recognition of nodal disease and subsequent consideration of regional treatment or systemic therapy. Prospective studies should therefore report the pattern, timing, treatment, morbidity and oncologic outcomes of regional recurrence after negative SLNB.

From a safety perspective, SLNB was associated with a low overall complication rate (3.2%), with most complications being minor or self-limited. This aligns with previous reports and supports the procedural safety of SLNB when performed in experienced hands. When using both radiocolloid and blue dye (or new tracers like ICG), SLNB morbidity is minimal compared to elective neck dissection or wide-field surgery.^18^

Importantly, despite plastic surgeons frequently managing high-risk cutaneous SCC, particularly of the head and neck and extremities, the majority of the available evidence identified in this review originates from the dermatologic and multidisciplinary oncology literature, with relatively limited representation from plastic surgery. This review therefore synthesizes evidence that has not previously been comprehensively presented from a plastic surgery perspective and highlights an important gap in specialty-specific evidence to guide reconstructive surgeons involved in the management of high-risk cSCC.

While this review provides important insights into SLNB use in cSCC, it is not without limitations. Most included studies were retrospective in nature and subject to selection and reporting bias, as clinicians may have preferentially offered SLNB to patients with more aggressive features. The absence of randomized control trials comparing SLNB with observation or elective nodal dissection also prevents definitive conclusions about the procedure's impact on long-term outcomes versus simpler, earlier disease detection. Furthermore, because our review focused exclusively on patients undergoing SLNB, we were unable to compare oncologic outcomes with similar high-risk cSCC patients managed without SLNB. Such comparative studies are needed to determine whether SLNB provides incremental prognostic or therapeutic benefit beyond conventional clinicopathologic risk stratification. Substantial variability in SLNB technique further clouds interpretation. Studies differed in tracer selection (radioisotopes, blue dyes, indocyanine green), injection protocols (peritumoral vs transdermal) and timing relative to tumour excision. Some centers combined biopsy and wide local excision in one session, while others staged them separately. These differences are likely to influence lymphatic mapping success and false negative rates. Similarly, pathology practices were not uniform and underreported. Variability also extended to how a ‘positive’ node was defined with approaches ranging from single-section hematoxylin and eosin staining to ultrastaging with serial cuts and immunohistochemistry, and few studies specified section thickness or centralized slide review. These inconsistencies introduce interstudy variability in reported positivity and false negative rates.

## Conclusion

In summary, our focused review confirms that SLNB is a reliable staging tool in non-mucosal cSCC, achieving a high node-detection rate (96.9%), with minimal morbidity. When SLNB is positive, it reliably flags tumours with aggressive features such as larger size, deeper invasion, poor differentiation and perineural spread. These patients face substantially higher risks of recurrence and disease-specific mortality. However, the 13.2% false-negative rate serves as a reminder that a negative SLNB cannot be taken as definitive proof of node-negative disease. Ongoing clinical surveillance remains essential, especially in tumours with established high-risk characteristics.

Going forward, prospective, multicenter studies with standardized SLNB protocols and pathology assessment methods are needed. These studies will help clarify which patients stand to benefit most from SLNB, minimize false negatives and ultimately determine whether early nodal detection can be translated into improved long-term outcomes. SLNB should currently be regarded as a helpful, though not definitive, approach to managing high-risk cSCC.

## Supplemental Material

sj-docx-1-psg-10.1177_22925503261491621 - Supplemental material for Diagnostic Accuracy and Predictive Factors of Sentinel Lymph Node Biopsy in Cutaneous Squamous Cell Carcinoma: A Systematic Review Supplemental material, sj-docx-1-psg-10.1177_22925503261491621 for Diagnostic Accuracy and Predictive Factors of Sentinel Lymph Node Biopsy in Cutaneous Squamous Cell Carcinoma: A Systematic Review by Omar El Sewify, Marion Sylvain, Cory Tremblay, Helene Retrouvey and Ammara Ghumman in Plastic Surgery / Chirurgie Plastique

sj-docx-2-psg-10.1177_22925503261491621 - Supplemental material for Diagnostic Accuracy and Predictive Factors of Sentinel Lymph Node Biopsy in Cutaneous Squamous Cell Carcinoma: A Systematic Review Supplemental material, sj-docx-2-psg-10.1177_22925503261491621 for Diagnostic Accuracy and Predictive Factors of Sentinel Lymph Node Biopsy in Cutaneous Squamous Cell Carcinoma: A Systematic Review by Omar El Sewify, Marion Sylvain, Cory Tremblay, Helene Retrouvey and Ammara Ghumman in Plastic Surgery / Chirurgie Plastique

sj-docx-3-psg-10.1177_22925503261491621 - Supplemental material for Diagnostic Accuracy and Predictive Factors of Sentinel Lymph Node Biopsy in Cutaneous Squamous Cell Carcinoma: A Systematic Review Supplemental material, sj-docx-3-psg-10.1177_22925503261491621 for Diagnostic Accuracy and Predictive Factors of Sentinel Lymph Node Biopsy in Cutaneous Squamous Cell Carcinoma: A Systematic Review by Omar El Sewify, Marion Sylvain, Cory Tremblay, Helene Retrouvey and Ammara Ghumman in Plastic Surgery / Chirurgie Plastique

sj-docx-4-psg-10.1177_22925503261491621 - Supplemental material for Diagnostic Accuracy and Predictive Factors of Sentinel Lymph Node Biopsy in Cutaneous Squamous Cell Carcinoma: A Systematic Review Supplemental material, sj-docx-4-psg-10.1177_22925503261491621 for Diagnostic Accuracy and Predictive Factors of Sentinel Lymph Node Biopsy in Cutaneous Squamous Cell Carcinoma: A Systematic Review by Omar El Sewify, Marion Sylvain, Cory Tremblay, Helene Retrouvey and Ammara Ghumman in Plastic Surgery / Chirurgie Plastique
