# Supplementary material for: Diagnostic Accuracy and Predictive Factors of Sentinel Lymph Node Biopsy in Cutaneous Squamous Cell Carcinoma: A Systematic Review
Source: Plast Surg (Oakv). 2026 Sep 30:22925503261491621. Online ahead of print. doi: 10.1177/22925503261491621 (PMC13627455; doi:10.1177/22925503261491621)
Supplement: Supplementary file 1 [file sj-docx-1-psg-10.1177_22925503261491621.docx]

**Supplemental Table S1. Study Characteristics**

| **Author** | **Article Title** | **Year** | **Country** | **Funding** | **Methodology** |
| --- | --- | --- | --- | --- | --- |
| Agrawal et al. | [99mTc] Tilmanocept Accurately Detects Sentinel Lymph Nodes and Predicts Pathologic Nodal Status in Patients with Squamous Cell Carcinoma of the Head and Neck: Results of a Phase 3 Multi-institutional Trial | 2014 | USA | Navidea Biopharmaceuticals, Dublin Ohio, USA | Multicenter, non-randomized, single-arm trial |
| Askari et al. | Squamous Cell Carcinoma of the Hand: A 20-Year Review | 2013 | USA | None | Single institution retrospective chart review |
| Chabrillac et al. | Cutaneous Squamous Cell Carcinoma Tumour Size is Associated with Sentinel Lymph Node Metastasis in a Cohort of 69 Patients | 2019 | France | NR | Bi-centric retrospective analysis |
| Chak et al. | A Positive Sentinel Lymph Node in Periocular Invasive Squamous Cell Carcinoma: A Case Series | 2013 | USA | None | Case series |
| Civantos et al. | Lymphatic Mapping and Sentinel Lymphadenectomy for 106 Head and Neck Lesions: Contrasts Between Oral Cavity and Cutaneous Malignancy | 2006 | USA | NR | Prospective series/study |
| Cuccia et al. | The Use of Sentinel Node Biopsy and Selective Lymphadenectomy in Squamous Cell Carcinoma of the Upper Limb. | 2008 | Italy | NR | Consecutive prospective series |
| Demir et al. | Sentinel lymph node biopsy with a gamma probe in patients with high-risk cutaneous squamous cell carcinoma: follow-up results of sentinel lymph node-negative patients | 2011 | Turkey | Scientific Project Support Unit of Kocaeli University | Prospective study |
| Dimonitsas et al. | Tracking the risk factors associated with high-risk cSCC: A 10-year, Two-Institution, Greek study | 2021 | Greece | Onassis Foundation-Scholarship (First author’s thesis) | Retrospective study |
| Durham et al. | Sentinel Lymph Node Biopsy for Cutaneous Squamous Cell Carcinoma on the Head and Neck | 2016 | USA | None | Retrospective review |
| Fujisawa et al. | Indocyanine Green Fluorescence-Navigated Sentinel Node Biopsy Showed Higher Sensitivity Than the Radioisotope or Blue Dye Method, Which May Help to Reduce False-Negative Cases in Skin Cancer | 2012 | Japan | National Cancer Center Research and Development Fund | Prospective study |
| Fujisawa et al. | Evaluation of positron emission tomography imaging to detect lymph node metastases in patients with high-risk cutaneous squamous cell carcinoma | 2016 | Japan | Grant-in-Aid for Scientific Research from the Japan Society for the Promotion of Science | Prospective study |
| Fujisawa et al. | Measurement of cutaneous lymphatic flow rates in patients with skin cancer: area extraction method. | 2014 | Japan | Grant-in-Aid for Scientific Research from the Japan Society for the Promotion of Science | Prospective study |
| Fujisawa et al. | Evaluation of sentinel node biopsy for cutaneous squamous cell carcinoma. | 2014 | Japan | Grant-in-Aid for Scientific Research from the Japan Society for the Promotion of Science and National Cancer Center Research Development Fund | Case series |
| Gore et al. | Prospective study of sentinel node biopsy for high-risk cutaneous squamous cell carcinoma of the head and neck | 2016 | Australia | NR | Prospective study |
| Hatta et al. | Implications of Popliteal Lymph Node Detected by Sentinel Lymph Node Biopsy | 2005 | Japan | Grant-in-Aid for Cancer Research from the Ministry of Health, Labor and Welfare | Prospective? |
| Ilmonen et al. | Sentinel lymph node biopsy in high-risk cutaneous squamous cell carcinoma of the head and neck | 2022 | Finland | Departmental sources | Retrospective study |
| Jankovic et al. | Lymphatic drainage map of the head and neck skin squamous cell carcinoma detected by sentinel lymph node biopsy | 2021 | Serbia | NR | Prospective analysis |
| Jansen et al. | The prognostic value of sentinel lymph nodes on distant metastasis–free survival in patients with high-risk squamous cell carcinoma | 2019 | Germany | None | Ambidirectional cohort study with prospective patient recruitment |
| Kofler et al. | Sentinel lymph node biopsy for high-thickness cutaneous squamous cell carcinoma | 2021 | Germany | None | Retrospective analysis |
| Krediet et al. | Sentinel lymph node biopsy and risk factors for predicting metastasis in cutaneous squamous cell carcinoma | 2015 | Germany | None | Retrospective analysis + Case series (mixed methods) |
| Kwon et al. | Sentinel lymph node biopsy for high-risk cutaneous squamous cell carcinoma: clinical experience and review of literature | 2011 | USA | NR | Retrospective case series |
| Lhote et al. | Sentinel Lymph Node Biopsy in Cutaneous Squamous Cell Carcinoma Series of 37 Cases and Systematic Review of the Literature | 2018 | France | NR | Multi-centre retrospective case series |
| Maeda et al. | Neutrophil-to-lymphocyte ratio is associated with survival and sentinel lymph node positivity in invasive cutaneous squamous cell carcinoma: A retrospective study | 2022 | Japan | None | Single-center retrospective case series |
| Maruyama et al. | Availability of sentinel lymph node biopsy for cutaneous squamous cell carcinoma. | 2017 | Japan | NR | Retrospective study |
| Matthey-Gie et al. | Sentinel Lymph Node Biopsy in Nonmelanoma Skin Cancer Patients | 2013 | Switzerland | NR | Case series |
| Mooney et al. | Sentinel Node Biopsy in 105 High-Risk Cutaneous SCCs of the Head and Neck: Results of a Multicenter Prospective Study | 2019 | Australia | None | Multicenter prospective study |
| Navarrete-Dechent et al. | Staging System Performance and Clinical Outcomes for Cutaneous Squamous Cell Carcinoma of the Ear: A Single-Center Retrospective Study | 2023 | USA | National Cancer Institute/National Institutes of Health | Retrospective study |
| Pollock et al. | Sentinel Node Lymphoscintigraphy in High-risk Cutaneous Squamous Cell Carcinoma | 2016 | USA | NR | Prospective analysis |
| Pride et al. | Outcomes of Sentinel Lymph Node Biopsy for Primary Cutaneous Squamous Cell Carcinoma of the Head and Neck | 2022 | USA | NR | Retrospective review |
| Rastrelli et al. | Sentinel Node Biopsy for High-Risk Cutaneous Nonanogenital Squamous Cell Carcinoma: A Preliminary  Result | 2010 | Italy | NR | Case series |
| Renzi et al. | Sentinel lymph node biopsy for high risk cutaneous squamous cell carcinoma: Case series and review of the literature | 2007 | Italy | Italian Ministry of Health | Case series |
| Reschly et al. | Utility of Sentinel Lymphadenectomy in the Management of Patients With High-Risk Cutaneous Squamous Cell Carcinoma | 2003 | USA | NR | Case series |
| Sahn and Lang | Sentinel Lymph Node Biopsy for High-Risk Nonmelanoma Skin Cancers | 2007 | USA | NR | Retrospective review |
| Samsanavicius et al. | Importance of sentinel lymphatic node biopsy in patients with low-risk and high-risk cutaneous squamous cell carcinoma | 2018 | Lithuania | None | Prospective study |
| Samsanavicius et al. | Sentinel lymph node biopsy for high-risk cutaneous squamous cell carcinoma: Analysis of recurrence-free survival | 2016 | Lithuania | None | Retrospective study |
| Spyropoulou et al. | Prospective Randomized Study on the Use of Sentinel Node Biopsy for High-risk Cutaneous Squamous Cell Carcinomas of the Head and Neck | 2024 | Greece | None | Prospective randomized study |
| Takahashi et al. | Sentinel node biopsy for high-risk cutaneous squamous cell carcinoma | 2014 | Japan | None | Retrospective analysis |
| Takeda et al. | Preoperative risk factors of lymph node metastasis in cutaneous squamous cell carcinoma | 2013 | Japan | NR | Retrospective study |
| Tejera-Vaquerizo et al. | Sentinel lymph node biopsy versus observation in high-risk cutaneous squamous cell carcinoma in immunosuppressed and immunocompetent patients: An inverse probability of treatment weighting study | 2024 | Spain | Grant from Fundacion Piel Sana of the Academia Espanola de Dermatologia y Venereologia | Multicenter, retrospective, observational cohort study |
| Tremblay-Abel et al. | Sentinel Lymph Node Biopsy in High-Risk Cutaneous Squamous Cell Carcinoma: Analysis of a Large Size Retrospective Series. | 2021 | Canada | NR | Retrospective case series |
| Wagner et al. | Sentinel Node Biopsy for High-Risk Nonmelanoma Cutaneous Malignancy | 2004 | USA | None | Consecutive case series |
| Wu et al. | Sentinel Lymph Node Biopsy for High-Risk Cutaneous Squamous Cell Carcinoma of the Head and Neck | 2020 | USA | None | Retrospective case series |
| Yasuta et al. | Usefulness of CT-lymphography in sentinel lymph node navigation | 2014 | Japan | NR | Prospective study |
| Zhang et al. | Sentinel lymph node biopsy versus observation in high risk cutaneous squamous cell carcinoma of head and neck: a propensity score matching analysis | 2024 | China | Henan provincial Medical Science and Technology Research Project and Project of Science and Technology in Science and Technology Department of Henan Province | Retrospective cohort study |

NR: Not Reported, USA: United States of America
