# Supplementary material for: Diagnostic Accuracy and Predictive Factors of Sentinel Lymph Node Biopsy in Cutaneous Squamous Cell Carcinoma: A Systematic Review
Source: Plast Surg (Oakv). 2026 Sep 30:22925503261491621. Online ahead of print. doi: 10.1177/22925503261491621 (PMC13627455; doi:10.1177/22925503261491621)
Supplement: Supplementary file 2 [file sj-docx-2-psg-10.1177_22925503261491621.docx]

**Supplemental Table S2. Patient Characteristics**

| **Study** | **N** | **Mean Age (range) (in years)** | **Sex** | **Comorbidities** |
| --- | --- | --- | --- | --- |
| Agrawal et al. (2014) | 4 | NR | NR | NR |
| Askari et al. (2013) | 86 | 69 (39-89) | M: 61  F: 25 | Prior nonskin cancer: 27  No previous cancer: 53  Prior cutaneous cancer: 31  Prior SCC: 42  Prior skin cancer or SCC: 53  Diabetes: 18  Heart condition: 12  Renal failure: 9  Tobacco use: 8  Prior transplant: 17 |
| Chabrillac et al. (2019) | 69 | 72 (39-89) | M: 61  F: 8 | Diabetes: 9/69  Immunodeficiency:  -HIV: 2/69  -Transplantation: 5/69  -Haematological malignancy: 5/69 |
| Chak et al. (2013) | 5 | 76.2 (51-92) | M: 4  F: 1 | Prior skin malignancies at another site: 1 |
| Civantos et al. (2006) | 11 | NR | NR | NR |
| Cuccia et al. (2008) | 6 | 74.5 (67-84) | M: 3  F: 3 | NR |
| Demir et al. (2011) | 14  (15 SCCs) | 71.5 (47-87) | M: 8  F: 6 | NR |
| Dimonitsas et al. (2021) | 36 | NR | NR | None immunocompromised |
| Durham et al. (2016) | 53  (54 SCCs) | 73 (47-90) | M: 44  F: 9 | 14 immunosuppressed  9 organ transplants  1 chronic lymphocytic leukemia  1 non-Hodgkin lymphoma  2 on immunosuppressants for ulcerative colitis and rheumatoid arthritis |
| Fujisawa et al. (2012) | 8  (9 SCCs) | NR | NR | NR |
| Fujisawa et al. (2016) | 26 | 68.1 (31-88) | M: 18  F: 8 | NR |
| Fujisawa et al. (2014) | 10 | 66.0 (31-86) | M: 6  F: 4 | NR |
| Fujisawa et al. (2014) | 54 | 69 (20-87) | M: 37  F: 17 | NR |
| Gore et al. (2016) | 57 | 67 (29-90) | M: 47  F: 10 | NR |
| Hatta et al. (2005) | 4 | 69.3 (54-80) | M: 4  F: 0 | NR |
| Ilmonen et al. (2022) | 63 | 71.1 (40.1-91) | M: 41  F: 22 | 17 immunosuppressed (4 kidney transplant, 1 liver transplant, 1 B-cell lymphoma, 1 Waldenstrom)  3 rheumatoid arthritis  1 sarcoidosis  1 polymyalgia rheumatica  1 Werner syndrome  1 epidermodysplasia  1 autoimmune hepatitis  1 discoid lupus erythematosus  1 multiple myeloma |
| Jankovic et al. (2021) | 64 | 71.5 (58-84) | M: 40  F: 24 | NR |
| Jansen et al. (2019) | 114 | 72.2 (42-89) | M: 89  F: 25 | 29 immunosuppressed |
| Kofler et al. (2021) | 150 (but only 101 with successful SLNB) | 71.8 (NR) | M: 126  F: 24 | 34 immunosuppressed |
| Krediet et al. (2015) | 143 | 73 (24-102) | M: 111  F: 32 | 35 immunosuppressed |
| Kwon et al. (2011) | 5 | 71.6 (51-89) | M: 4  F: 1 | NR |
| Lhote et al. (2018) | 37 | NR | M: 30  F: 7 | 10 immunosuppressed (6 haematological malignancy, 4 organ transplant) |
| Maeda et al. (2022) | 50 | Mean 1: 69.9 (n=10)  Mean 2: 70.8 (n=40)  (NR) | M: 29  F: 21 | NR |
| Maruyama et al. (2017) | 49 | 66 (30-86) | M: 31  F: 18 | 1 osteomyelitis  1 radiation dermatitis  2 discoid lupus erythematosus  2 lichen sclerosis et atrophicus  1 epidermolysis bullosa dystrophic recessive  1 Muir-Torre syndrome |
| Matthey-Gie et al. (2013) | 7 | 66.57 (39-92) | M: 4  F: 3 | 1 epidermolisis bullosa dystrophyca |
| Mooney et al. (2019) | 104 | 65 (27-90) | M: 90  F: 14 | NR |
| Navarrete-Dechent et al. (2023) | 30 | NR | NR | NR |
| Pollock et al. (2016) | 22 | 67.09 (44-80) | NR | NR |
| Pride et al. (2022) | 58 | 67 (15-90) | M: 48  F: 10 | 25 immunosuppressed (10 renal transplant, 2 renal and pancreatic transplant, 3 lung transplant, 2 rheumatoid arthritis, 2 Hodgkin lymphoma, 2 chronic lymphocytic leukemia, 1 renal and lung transplant, 3 others) |
| Rastrelli et al. (2012) | 19  (20 SCCs) | 71.9 (49-90) | NR | NR |
| Renzi et al. (2007) | 22 | 64.2 (35-80) | M: 19  F: 3 | None immunocompromised |
| Reschly et al. (2003) | 7 | 60.86 (41-76) | M: 4  F: 3 | NR |
| Sahn and Lang (2007) | 8  (9 SCCs) | 67.38 (52-84) | M: 8  F: 0 | 4 immunosuppressed (1 severe psoriasis, 3 organs transplants) |
| Samsanavicius et al. (2018) | 88 | Mean 1: 73.38 (n=42)  Mean 2: 75.98 (n=46)  (NR) | M: 33  F: 55 | NR |
| Samsanavicius et al. (2016) | 51 | Mean male: 76.29 (66-85) (n=17)  Mean female: 74.87 (48.61-92.52) (n=34) | M: 17  F: 34 | NR |
| Spyropoulou et al. (2024) | 37 | 74.08 (NR) | M: 33  F: 4 | Smoker: 15  Non-smoker: 22 |
| Takahashi et al. (2014) | 19 | 69.74 (47-88) | M: 10  F: 9 | NR |
| Takeda et al. (2013) | 21 | NR | NR | NR |
| Tejera-Vaquerizo et al. (2024) | 70 | NR | M: 54  F: 16 | 42 immunocompetent  28 immunosuppressed |
| Tremblay-Abel et al. (2021) | 93  (94 SCCs) | 76 (32-95) | M: 76  F: 14 | 23 immunosuppressed (10 organ transplant, 8 hematologic cancer, 4 inflammatory or autoimmune disease, 1 HIV) |
| Wagner et al. (2004) | 11 | 61.1 (32-93) | NR | NR |
| Wu et al. (2020) | 84 (but 1 did not undergo SLNB due to occult metastasis intraoperatively) | 69 (17-89) | M: 68  F: 16 | 28/84 immunosuppressed  (13 solid organ transplant, 11 lymphoma/leukemia/myeloma, 1 myelofibrosis, 1 rheumatoid arthritis, 1 discoid lupus, 1 xeroderma pigmentosum)  History of multiple cSCCs: 44/84 |
| Yasuta et al. (2014) | 5 | 73.4 (68-85) | M: 3  F: 2 | NR |
| Zhang et al. (2024) | 1169 | NR | M: 806  F: 363 | NR |

cSCC: cutaneous squamous cell carcinoma, F: female, HIV: human immunodeficiency virus, M: male, N: number of patients, NR: not reported, SCC: squamous cell carcinoma, SLNB: sentinel lymph node biopsy
