# Supplementary material for: Diagnostic Accuracy and Predictive Factors of Sentinel Lymph Node Biopsy in Cutaneous Squamous Cell Carcinoma: A Systematic Review
Source: Plast Surg (Oakv). 2026 Sep 30:22925503261491621. Online ahead of print. doi: 10.1177/22925503261491621 (PMC13627455; doi:10.1177/22925503261491621)
Supplement: Supplementary file 3 [file sj-docx-3-psg-10.1177_22925503261491621.docx]

**Supplemental Table S3. Tumour Characteristics**

| **Study** | **Location** | **Diameter** | **Depth** | **Recurrence** | **High-risk** | **Differentiation** | **Chronic Inflammation** | **Invasion** |
| --- | --- | --- | --- | --- | --- | --- | --- | --- |
| Agrawal et al. (2014) | NR | NR | NR | NR | NR | NR | NR | NR |
| Askari et al. (2013) | Hand: 72 Digits: 10 Web space: 6 Wrist: 5 | N=76 <101 mm^2: 37 101-500: 30 >500: 9 | N=15 ≤2mm: 8 ≥2mm: 7 | History of prior recurrence: 12 | NR | N=63 G0: 4 (in-situ) G1: 47 (WD) G2: 9 (MD) G3: 3 (PD) | NR | NR |
| Chabrillac et al. (2019) | Lip: 34.8% External ear: 14.5% Peri-auricular: 10.1%  Head&Neck: 92.8% Others in sun-exposed areas | Median: 25mm (2-110mm range) -≤20mm: 31/69 - >20mm: 34/69 - 4/69 NR | n=33 Median: 6.5mm (range 2-20mm) Clark's level: - II: 1/33 - III: 1/33 - IV: 3/33 - V: 28/33 | 15/69 | All high risk | WD: 37/69 MD: 15/69 PD: 8/69 Sarcomatoid: 2/69 | NR | 17/69 PNI |
| Chak et al. (2013) | 2 lower eyelid 2 upper eyelid 1 lower eyelid + cheek + anterior orbital extension | Mean: 3.56cm (n=5) [4-2.5-4.3-4-3] | NR | 0/5 | All high risk | NR | 4/5 Ulceration | NR |
| Civantos et al. (2006) | 4 cheek 1 nasal vestibule 1 inferior neck 1 auricle+preauricle 1 brow+temple 1 temple 1 auricle 1 Extensive facial CA | NR | NR | 0/11 | All high risk | NR | NR | NR |
| Cuccia et al. (2008) | 6 arm | Mean: 3.45cm (range 2.25-4.6)cm | NR | NR | NR | WD: 1/6 MD: 3/6 UD: 1/6 NR: 1/6 | 3/6 Ulceration | 2/6 PNI |
| Demir et al. (2011) | 1 Popliteal 1 temporal scalp 2 hand dorsum 3 auricular 1 nose dorsum 1 frontoparietal + cervical 1 femoral 1 web of 3rd toe 1 preauricular 1 frontal 1 vertex scalp (n=15 SCCs) | Mean: 5.33cm (range 0.5-20) (n=15 SCCs) | All <4mm | 0/15 SCCs | All high risk | WD: 11/15 MD: 2/15 PD: 2/15 (n=15 SCCs) | NR | 0/15 PNI (SCCs) |
| Dimonitsas et al. (2021) | Abdomen, lip, upper arm, and knee | NR | NR | NR | All high risk | NR | 0/36 Ulceration | 0 LVI |
| Durham et al. (2016) | 24 cheek or forehead 14 scalp 9 ear 4 lip 2 neck 1 nose | Mean 2.56cm | NR | 6 SCCs | All pts high-risk SCC 1 at radiation site, 1 at chronic ulcer site | Initial diagnostic biopsy: WD: 10/54  MD: 23/54 PD: 15/54 Sarcomatoid: 2/54 NR: 4/54 (n=54 SCCs)  WLE: WD: 4/54 MD: 26/54 PD: 18/54 Sarcomatoid: 2/54 NR: 4/54 | 1 radiation area  1 chronic ulcer | 19/54 PNI (SCCs)  5/54 LVI (SCCs) |
| Fujisawa et al. (2012) | 1 back 1 fifth toe 1 lower leg 2 forearm 1 poplitea 1 second finger 1 foot | NR | NR | NR | All high risk | NR | NR | NR |
| Fujisawa et al. (2016) | 2 auricle 1 preauricular 1 little finger 2 parietal 1 upper arm 3 dorsal hand 2 sole 1 temporal 1 occipital 1 cheek 1 mandibular 1 forearm 1 palm 1 index 1 middle finger 1 median ant chest 1 lower abdomen 1 sacral 1 lower leg 1 ankle 1 lower lip | Mean: 37.8mm (range 5-95)mm | Mean: 9.6mm (range 2.0-35mm) | NR | All high risk | WD: 17/26 MD: 7/26 PD: 2/26 | NR | 4/26 PNI |
| Fujisawa et al. (2014) | 1 parietal 1 occipital 1 median ant chest 1 lower abdomen 1 upper arm 1 forearm 1 palm 1 dorsal hand 1 index 1 sole | NR | NR | NR | NR | NR | NR | NR |
| Fujisawa et al. (2014) | 25 head and neck 13 upper limb 12 lower limb 2 trunk 2 genital area | NR | NR | NR | NR | NR | NR | NR |
| Gore et al. (2016) | 14 scalp 8 forehead/temple 6 ear 7 cheek 6 nose 12 lip 4 neck | Mean: 25mm (range 6-65mm) <20mm: 21 20-50mm: 32 ≥50mm: 4 | Mean 9.2mm (range 1.0-22mm) 0-5mm: 19 >5mm: 38 | 17/57 | All high risk | WD: 10/57 MD: 27/57 UD: 20/57 | NR | 22/57 PNI  3/57 LVI |
| Hatta et al. (2005) | 1 lower leg 1 lateral malleolus 1 dorsal foot 1 heel | NR | NR | NR | NR | NR | NR | NR |
| Ilmonen et al. (2022) | 25 lower lip 6 upper lip 10 ear 8 forehead 8 scalp 6 cheek | Mean 16.4mm (3-40), median 15mm | Mean 6.7mm (2.2-31mm), median 6mm | NR | All high risk | WD: 29/63 MD: 30/63 UD: 4/63 | NR | 12/63 PNI |
| Jankovic et al. (2021) | NR | NR | NR | NR | NR | NR | NR | NR |
| Jansen et al. (2019) | 87 head and neck 27 trunk | NR | Mean 6.2mm (2.8-18) | NR | All high risk | 14 undifferentiated | 47 Ulceration | 16 PNI |
| Kofler et al. (2021) | NR | Mean 35.05mm (n=150) | Mean 8.72mm (n=150) | NR | All high risk | NR | NR | NR |
| Krediet et al. (2015) | 10 upper limb 18 lower limb 9 trunk 14 ear 14 lip 6 nose  72 rest of head | Mean 4.6 (cm) (range 0.1-100) | Mean 3.0mm (range 0.5-8) | NR | 58 high risk  85 non high risk | WD: 26/143 MD: 23/143 PD: 6/143 NA: 88/143 | NR | NR |
| Kwon et al. (2011) | 1 forearm 1 chest wall 2 temporal 1 upper arm | Mean 3.2cm (1.3-7cm)  ≥20mm (trunk/extremities): 2 ≥10mm (head): 0 ≥6mm (face/genitalia): 2 <20mm (arm): 1 | NR | 3/5 | All high risk | WD: 2/5 MD or PD: 3/5 | NR | 1/5 PNI |
| Lhote et al. (2018) | NR | >2cm: 29/37 | >4mm: 22/37 | 0/37 | All high risk | PD: 4/37 | NR | 5/37 PNI  2/37 LVI |
| Maeda et al. (2022) | NR | NR | NR | NR | NR | NR | NR | NR |
| Maruyama et al. (2017) | 11 head and neck 1 trunk 6 genitals and anus 11 upper extremity 20 lower extremity | NR | NR | NR | All high risk | NR | 6 scars  1 osteomyelitis  1 radiation dermititis | NR |
| Matthey-Gie et al. (2013) | 3 leg 1 thumb 1 thigh 1 forearm 1 hand | NR | NR | 1/7 | All high-risk | 1 PD | NR | NR |
| Mooney et al. (2019) | 18 scalp 18 forehead/temple 12 ear 16 cheek 8 nose 24 lip 9 neck (n=105 lesions, 104 pts) | Mean 23.3mm (2.9-65, n=101 lesions) <20mm: 45 20-50mm: 50 >50mm: 6 NR: 4 (n=105 lesions, 104 pts) | Mean 8.5mm (1-25mm, n=101 lesions) 0-5mm: 27 >5mm: 74 NR: 4 (n=105 lesions, 104 pts) | 33/105 lesions (104 pts) | All high risk | WD: 21/105 MD: 38/105 PD: 45/105 NR: 1/105 | NR | 42/105 PNI (SCCs)  7/105 LVI (SCCs) |
| Navarrete-Dechent et al. (2023) | 30 external ear | NR | NR | NR | All high risk | NR | NR | NR |
| Pollock et al. (2016) | 1 fifth finger 1 fourth toe 2 ear 3 hand 1 posterior neck 1 lower leg 2 cheek 1 forearm 3 forehead 1 thigh 1 AKA 1 index 2 nose 1 leg 1 temple | Mean 2.985cm (n=20)  <2cm: 8 ≥2cm: 14 | Mean 5.845mm (n=20) | 4/22 | All high risk | WD: 9/22 MD: 3/22 PD: 9/22 NR: 1/22 | NR | 3/22 PNI  0/22 LVI |
| Pride et al. (2022) | All head and neck | NR | NR | NR | NR | NR | NR | NR |
| Rastrelli et al. (2012) | 2 temporal 2 nose 1 neck 1 cheek 2 occipital 1 chin 1 ear 1 leg 1 forearm 1 chest 1 hallux 3 foot 1 heel 1 leg+arm | Mean 1.89cm (0.5-6) (n=15pts, 16 lesions) | Mean 3.58mm (0.7-9.6) (n=17 lesions, 16 pts) | NR | All high risk | WD: 4/20 lesions (4/19 pts) MD: 12/20 lesions (11/19 pts) PD: 3/20 lesions (3/19 pts) NR: 1/20 lesions (1/19 pts) | NR | 1/20 PNI (SCCs, 1/19 pts) (16/20 no, 3/10 NR) |
| Renzi et al. (2007) | 13 lower lip or lip commissure 2 ear 7 NR | Mean 2.7cm (1.0-6.7)  14/22 had >2cm | 10/22 were >4mm | 3/22 | All high risk | 10/22 MD or PD | NR | 2/22 PNI |
| Reschly et al. (2003) | 1 wrist  2 anterior tibia 1 clavicule 1 scalp 1 AC fossa 1 helix | Mean 4.73cm (1-11) | Mean 5.44mm (1.4-10.4) | 1/9 | All high risk | WD: 4/7 MD: 1/7 PD: 2/7 | NR | 0/7 PNI  0/7 LVI |
| Sahn and Lang (2007) | 1 forehead 1 zygoma 1 ear 2 thumb 1 chest 1 mid back 2. leg (same pt) | Mean 7.33cm (3.0-15) (n=9 SCCs, 8 pts) | All Clark Level V | 3/8 pts (3/9 lesions) | All high risk | 7 MD/WD (6 pts) 1 MD/PD 1 WD | NR | 4/8 PNI (4/9 SCCs)  3/8 LVI (3/9 SCCs) |
| Samsanavicius et al. (2018) | 58 head and neck 6 torso 12 arm 12 leg | Mean 0.97cm (n=42) Mean 2.25cm (n=46) | Mean 1.39mm (n=42) Mean 2.97mm (n=46) | NR | 46 high risk 42 low risk | 42 WD/MD 46 PD/UD | 48/88 Ulceration | NR |
| Samsanavicius et al. (2016) | 33 head and neck 9 lower extremity 6 trunk 3 upper extremity | Mean men: 2.18cm (0.4-25) (n=17) Mean women: 2.23cm (0.5-30) (n=34) | Mean men: 2.5mm (n=17) Mean women: 2.07mm (n=34) | NR | 31 high-risk 20 low-risk | WD: 37/51 MD/PD: 11/51 UD: 3/51 | NR | NR |
| Spyropoulou et al. (2024) | 5 external ear 12 lower lip 11 scalp 6 cheek 1 nose 1 forehead | ≤2cm: 13 2-5cm: 17 ≥5cm: 7 | Breslow: ≤3.5mm: 5 >3.5mm: 32 | NR | All high-risk | WDl: 7 WD/MD: 4 MD: 16 MD/PD: 7 PD: 3 | NR | 6/37 PNI |
| Takahashi et al. (2014) | 5 head and neck 7 lower extremity 5 upper extremity 2 trunk | Mean 4.08cm (1.8-8) | Mean 6.49mm (1.4-24.8) | NR | All high risk | WD: 15 MD: 3 PD: 1 | 7/19 radiation or chronic inflammatory process | NR |
| Takeda et al. (2013) | NR | NR | NR | NR | NR | NR | NR | NR |
| Tejera-Vaquerizo et al. (2024) | 4 hand/foot 53 head and neck 13 trunk and extremities | Median 30.0mm immunocompetent Median 20.5mm immunosuppressed | Median 8.0mm immunocompetent Median 7.0mm immunosuppressed | NR | All high risk | WD: 12 MD: 40 PD: 17 NR: 1 (n=69) | NR | 19/70 PNI |
| Tremblay-Abel et al. (2021) | 33 scalp 14 ear 11 forehead 9 members 8 temple 7 nose 7 cheek 2 lip 2 neck 1 trunk (n=94 SCCs) | <2cm: 6 2-4cm: 44 ≥4cm: 44 (n=94 SCCs) | ≤6mm: 11 >6mm or beyond subcut fat: 83 (n=94 SCCs) | 12/70 | All high risk | WD: 25 MD: 46 PD: 19 UD: 2 NR: 2 | NR | 28/94 PNI (SCCs)  8/94 LVI (SCCs) |
| Wagner et al. (2004) | 3 face 1 hand 2 scalp 2 leg 1 ankle 2 arm | Mean 4.95cm (3-13) | NR | NR | All high-risk | NR | NR | NR |
| Wu et al. (2020) | 23 scalp 1 forehead 13 cheek 17 ear 2 neck 7 nose 4 periocular 10 lip 5 temple 2 postauricular (n=84) | <2cm: 23 ≥2 and <4cm: 41 ≥4cm: 16 NR: 4 | ≤6mm: 20 >6mm or deep subcutaneous fat: 29 NR: 35 | 21/84 | All high risk | WD to MD: 45 PD: 30 NS: 9 | 11/84 chronic inflammation | 35/84 PNI (21/84 no, 28/84 NR) |
| Yasuta et al. (2014) | 3 face 1 foot 1 vertex | NR | NR | NR | NR | NR | NR | NR |
| Zhang et al. (2024) | 40 temple/forehead 887 face/periorbital 189 scalp/neck 53 ear | NR | NR | NR | All high risk | WD: 148 MD: 145 PD: 876 | NR | 536/1169 PNI |

AC: acromioclavicular, AKA: above knee amputation, cm: centimeter, LVI: lymphovascular invasion, MD: moderately differentiated, mm: millimeter, N: number of tumours, NR: not reported, PD: poorly differentiated, PNI: perineural invasion, pts: patients, SCC: squamous cell carcinoma, UD: undifferentiated, WD: well differentiated
