# Supplementary material for: Diagnostic Accuracy and Predictive Factors of Sentinel Lymph Node Biopsy in Cutaneous Squamous Cell Carcinoma: A Systematic Review
Source: Plast Surg (Oakv). 2026 Sep 30:22925503261491621. Online ahead of print. doi: 10.1177/22925503261491621 (PMC13627455; doi:10.1177/22925503261491621)
Supplement: Supplementary file 4 [file sj-docx-4-psg-10.1177_22925503261491621.docx]

**Supplemental Table S4.**

Table A.

RoB2 (Risk of Bias Tool – Version 2) – Assessment of Randomized Controlled Studies

| Study | Risk of bias from randomization | Risk of bias due to deviations from intended interventions | Risk of bias due to missing outcome data | Risk of bias in measurement of the outcome | Risk of bias in selection of the reported result | Overall risk of bias |
| --- | --- | --- | --- | --- | --- | --- |
| Spyropoulou et al. (2024) | Some concerns | High risk | Low risk | Some concerns | Low risk | High risk |

Table B.

MINORs (Methodological Index for Non-Randomized Studies) Evaluation – For Comparative Studies (Maximum Score: 24)

| Study | Clearly stated aim | Inclusion of consecutive patients | Prospective collection of data | Endpoints appropriate to the aim of the study | Unbiased assessment of study endpoint | Follow-up period appropriate to aim of study | Loss to follow-up <5% | Prospective calculation of study size | Adequate control group | Contemporary groups | Baseline equivalence of groups | Adequate statistical analysis | Total |
| --- | --- | --- | --- | --- | --- | --- | --- | --- | --- | --- | --- | --- | --- |
| Chabrillac et al. (2019) | 2 | 2 | 0 | 2 | 2 | 2 | 2 | 0 | 2 | 2 | 1 | 2 | 19 |
| Dimonitsas et al. (2021) | 2 | 1 | 0 | 2 | 2 | 2 | 0 | 0 | 2 | 2 | 1 | 2 | 16 |
| Gore et al. (2016) | 2 | 1 | 2 | 2 | 2 | 1 | 2 | 2 | 2 | 2 | 1 | 2 | 21 |
| Jansen et al. (2019) | 2 | 1 | 0 | 2 | 2 | 2 | 2 | 0 | 2 | 2 | 1 | 2 | 18 |
| Kofler et al. (2021) | 2 | 1 | 0 | 2 | 1 | 2 | 1 | 0 | 2 | 2 | 1 | 2 | 16 |
| Krediet et al. (2015) | 2 | 1 | 0 | 2 | 2 | 2 | 1 | 0 | 2 | 2 | 1 | 2 | 17 |
| Lhote et al. (2018) | 2 | 1 | 0 | 2 | 2 | 1 | 1 | 0 | 2 | 2 | 1 | 2 | 16 |
| Maeda et al. (2022) | 2 | 1 | 0 | 2 | 2 | 2 | 1 | 0 | 2 | 2 | 1 | 2 | 17 |
| Maruyama et al. (2017) | 1 | 1 | 0 | 2 | 1 | 1 | 1 | 0 | 2 | 2 | 1 | 2 | 14 |
| Mooney et al. (2019) | 2 | 1 | 2 | 2 | 2 | 1 | 1 | 2 | 2 | 2 | 1 | 2 | 20 |
| Navarrete-Dechent et al. (2023) | 2 | 1 | 0 | 2 | 2 | 2 | 1 | 0 | 2 | 2 | 1 | 2 | 17 |
| Samsanavicius et al. (2018) | 2 | 1 | 2 | 2 | 2 | 2 | 2 | 0 | 2 | 2 | 2 | 2 | 20 |
| Tejera-Vaquerizo et al. (2024) | 2 | 0 | 0 | 2 | 2 | 2 | 0 | 0 | 2 | 2 | 2 | 2 | 18 |
| Zhang et al. (2024) | 2 | 1 | 0 | 2 | 2 | 2 | 1 | 0 | 2 | 2 | 2 | 2 | 21 |

Table B.

MINORs (Methodological Index for Non-Randomized Studies) Evaluation – For Non-Comparative Studies (Maximum Score: 16)

| Study | Clearly stated aim | Inclusion of consecutive patients | Prospective collection of data | Endpoints appropriate to the aim of the study | Unbiased assessment of study endpoint | Follow-up period appropriate to aim of study | Loss to follow-up <5% | Prospective calculation of study size | Total |
| --- | --- | --- | --- | --- | --- | --- | --- | --- | --- |
| Agrawal et al. (2014) | 2 | 1 | 2 | 2 | 2 | 2 | 2 | 2 | 15 |
| Askari et al. (2013) | 2 | 2 | 0 | 2 | 1 | 2 | 1 | 0 | 8 |
| Chak et al. (2013) | 2 | 0 | 0 | 2 | 2 | 1 | 2 | 0 | 9 |
| Civantos et al. (2006) | 2 | 1 | 2 | 2 | 2 | 2 | 1 | 0 | 12 |
| Cuccia et al. (2008) | 2 | 2 | 2 | 2 | 2 | 2 | 2 | 0 | 14 |
| Demir et al. (2011) | 2 | 0 | 2 | 2 | 2 | 2 | 2 | 0 | 12 |
| Durham et al. (2016) | 2 | 1 | 0 | 2 | 2 | 1 | 0 | 0 | 8 |
| Fujisawa et al. (2012) | 2 | 1 | 2 | 2 | 2 | 2 | 2 | 0 | 13 |
| Fujisawa et al. (2016) | 2 | 0 | 2 | 2 | 2 | 0 | 0 | 0 | 8 |
| Fujisawa et al. (2014) | 2 | 0 | 2 | 2 | 2 | 0 | 0 | 0 | 8 |
| Fujisawa et al. (2014) | 1 | 1 | 0 | 2 | 2 | 1 | 2 | 0 | 9 |
| Hatta et al. (2005) | 1 | 0 | 0 | 2 | 1 | 2 | 0 | 0 | 6 |
| Ilmonen et al. (2022) | 1 | 1 | 0 | 2 | 2 | 2 | 0 | 0 | 8 |
| Jankovic et al. (2021) | 1 | 0 | 0 | 2 | 2 | 2 | 0 | 0 | 7 |
| Kwon et al. (2011) | 1 | 0 | 0 | 2 | 2 | 1 | 2 | 0 | 8 |
| Matthey-Gie et al. (2013) | 1 | 0 | 0 | 2 | 1 | 1 | 2 | 0 | 7 |
| Pollock et al. (2016) | 2 | 1 | 2 | 2 | 2 | 2 | 2 | 0 | 12 |
| Pride et al. (2022) | 2 | 1 | 0 | 2 | 2 | 2 | 1 | 0 | 10 |
| Rastrelli et al. (2012) | 1 | 0 | 0 | 2 | 1 | 1 | 2 | 0 | 7 |
| Renzi et al. (2007) | 1 | 0 | 0 | 2 | 1 | 2 | 2 | 0 | 8 |
| Reschly et al. (2003) | 1 | 0 | 0 | 2 | 2 | 2 | 2 | 0 | 9 |
| Sahn and Lang (2007) | 1 | 0 | 0 | 2 | 1 | 1 | 2 | 0 | 7 |
| Samsanavicius et al. (2016) | 1 | 1 | 0 | 2 | 1 | 1 | 1 | 0 | 7 |
| Takahashi et al. (2014) | 1 | 1 | 0 | 2 | 2 | 1 | 2 | 0 | 9 |
| Takeda et al. (2013) | 1 | 1 | 0 | 2 | 1 | 1 | 1 | 0 | 7 |
| Tremblay-Abel et al. (2021) | 2 | 2 | 0 | 2 | 2 | 1 | 0 | 0 | 9 |
| Wagner et al. (2004) | 1 | 2 | 0 | 2 | 1 | 1 | 2 | 0 | 9 |
| Wu et al. (2020) | 2 | 1 | 0 | 2 | 2 | 1 | 1 | 0 | 9 |
| Yasuta et al. (2014) | 1 | 0 | 2 | 2 | 1 | 2 | 2 | 0 | 10 |
